# Gynecological laparoscopic surgery in Pokhara: four years’ experience at Charak memorial hospital

**DOI:** 10.1186/s12905-026-04594-w

**Published:** 2026-06-11

**Authors:** Bishnu Palikhe, Kovid Nepal, Om Kumar Shrestha, Manoj Tripathee, Pankaj Baral, Anup Shrestha, Prakash Sharma

**Affiliations:** 1 Department of Obstetrics and Gynecology, Charak Memorial Hospital, Pokhara, 33700 Nepal; 2 Department of Surgery, Charak Memorial Hospital, Pokhara, 33700 Nepal; 3 Department of Radiology, Charak Memorial Hospital, Pokhara, 33700 Nepal; 4 Department of Anaesthesia, Charak Memorial Hospital, Pokhara, 33700 Nepal; 5 Department of Radiology, Manipal College of Medical Sciences, Pokhara, 33700 Nepal

**Keywords:** Diagnostic laparoscopy, Minimal Invasive Surgery, Total Laparoscopic Hysterectomy

## Abstract

**Background:**

Minimally invasive surgery (MIS) has transformed gynecological practice worldwide because of its advantages including reduced postoperative pain, shorter hospital stay, and faster recovery. However, evidence regarding the implementation and outcomes of gynecological laparoscopy in low- and middle-income countries remains limited. This study aimed to evaluate the feasibility, safety, and perioperative outcomes of gynecological laparoscopic surgeries performed in a resource-constrained tertiary care center in Nepal.

**Methods:**

A retrospective descriptive study with limited comparative analysis was conducted at Charak Memorial Hospital, Pokhara, Nepal. Medical records of patients undergoing gynecological laparoscopic surgery between 2019 and 2023 were reviewed. Primary outcomes included conversion to laparotomy and perioperative complication rates. Secondary outcomes included operative time, intraoperative blood loss, and duration of hospital stay. Complications were classified according to the Clavien–Dindo classification system.

**Results:**

A total of 159 patients were planned for laparoscopic gynecological surgery during the study period. Among them, 153 procedures (96.2%) were completed laparoscopically, while 6 cases (3.8%) required conversion to laparotomy. The mean age of patients was 37.2 ± 12.1 years. Ovarian cystectomy was the most common procedure performed (62.1%), followed by total laparoscopic hysterectomy (26.8%). Histopathological examination of ovarian cystectomy specimens demonstrated predominantly benign lesions with no malignant pathology identified. Total laparoscopic hysterectomy had significantly longer operative time and greater intraoperative blood loss compared with other procedures (*P* < 0.001). Most patients (84.9%) were discharged within two days of surgery. The overall complication rate was 6.5%, with most complications classified as minor (Clavien–Dindo Grade I–II). Fever was the most common postoperative complication. One major complication involving pelvic vein thrombosis and one intraoperative urinary bladder injury were encountered.

**Conclusion:**

Gynecological laparoscopic surgery can be performed safely and feasibly in a resource-constrained setting with acceptable conversion and complication rates. Despite challenges related to resource limitations and the learning curve associated with MIS implementation, favorable perioperative outcomes were achieved. Further prospective and comparative studies are required to strengthen the evidence for wider adoption of minimally invasive gynecological surgery in similar low-resource settings.

## Introduction

In recent years, minimal invasive gynecological surgeries (MIS) have emerged as a cornerstone in modern surgical practices, transforming the landscape of women’s healthcare. With their myriad benefits such as minimized postoperative discomfort, shorter hospital stays. MIS techniques have gained widespread acclaim among both patients and medical professionals [1–4]. However, despite their global recognition, there remains a scarcity of literature delineating the complexity and outcomes of such procedures within the context of Nepal.

In light of lack of information, this study endeavors to bridge the existing gap by conducting a comprehensive retrospective analysis of laparoscopic gynecological surgeries at Charak Memorial Hospital (CMH) in Pokhara, Nepal, spanning a robust four-year timeframe. By studying the experiences and outcomes witnessed at CMH, this research aims to furnish valuable insights into the feasibility, safety and surgical outcomes of laparoscopic interventions in the Nepalese healthcare milieu.

Through meticulous examination of patient records, surgical techniques, and postoperative trajectories, this study endeavors to shed light on the evolving landscape of gynecological care in Pokhara, while also offering tangible contributions to the broader discourse on MIS practices in resource-constrained settings. By elucidating the challenges, triumphs, and lessons gleaned from the four-year experience at CMH, this research seeks to inform and enrich future endeavors aimed at optimizing women’s health outcomes in Nepal and beyond.

The primary objective of this study is to evaluate the feasibility and safety of gynecological laparoscopic surgery in a resource-constrained setting, using conversion rate and perioperative complication rate as primary outcomes. Secondary outcomes include operative time, intraoperative blood loss, and duration of hospital stay.

Although the advantages of minimally invasive surgery are well established in high-resource settings, there remains a relative paucity of data from low- and middle-income countries (LMICs), where healthcare systems face unique infrastructural, financial, and training-related challenges. Understanding the real-world implementation, outcomes, and limitations of MIS in such environments is essential for informing sustainable expansion of these techniques. This study therefore aims to provide a contextualized analysis of laparoscopic gynecological surgery in a resource-constrained tertiary care center in Nepal. To evaluate the feasibility and safety of gynecological laparoscopic surgery by analyzing operative characteristics, conversion rates, and perioperative complications in a resource-constrained setting.

## Methods

A retrospective descriptive study with limited comparative analysis was conducted at Charak Memorial Hospital (CMH), Pokhara, Nepal. The study included patients undergoing gynecological laparoscopic surgery between 2019 and 2023.

### Inclusion and exclusion criteria

Inclusion criteria included all patients scheduled for laparoscopic gynecological surgery during the study period. Patients with suspected malignancy, hemodynamic instability requiring primary laparotomy, or incomplete medical records were excluded.

### Surgical decision-making

The decision to perform laparoscopic surgery was based on clinical evaluation, ultrasonographic findings suggestive of benign pathology, hemodynamic stability of the patient, absence of suspicion of malignancy, and availability of laparoscopic expertise and equipment. In cases of ectopic pregnancy, only hemodynamically stable patients with mild to moderate hemoperitoneum were selected for laparoscopy, while unstable patients were managed by laparotomy.

### Definitions and outcome measures

Operative time was defined as the duration from skin incision to closure. Blood loss was estimated intraoperatively based on suction volume after subtracting irrigation fluid and visual estimation. Conversion to laparotomy was defined as the need to switch from laparoscopy to open surgery after initiation of the procedure.

Primary outcomes included conversion rate and perioperative complication rate. Secondary outcomes included operative time, intraoperative blood loss, postoperative recovery parameters, and duration of hospital stay.

Postoperative recovery parameters included time to oral intake, mobilization, and length of hospital stay. Complications were categorized as intraoperative and postoperative and graded according to the Clavien–Dindo classification system.

### Statistical analysis

Statistical analysis was performed using the Statistical Package for the Social Sciences (SPSS) version 29. Descriptive statistics were used to summarize patient characteristics and surgical outcomes. Continuous variables were expressed as mean ± standard deviation. One-way ANOVA was used for exploratory comparison of operative time and blood loss across different procedure types. Given the heterogeneity of procedures, these comparisons are interpreted cautiously and primarily for descriptive purposes.

## Results

Over the four-year study period, a total of 159 patients were planned for gynecological laparoscopic surgery. Among them, 153 procedures (96.2%) were successfully completed laparoscopically, while 6 cases (3.8%) required conversion to laparotomy due to surgical complexities. Conversion to laparotomy was included as a key outcome measure in the analysis.

Age of the patients ranged from 14 to 74 years, with a mean age of 37.2 ± 12.1 years. Most patients were in the age group of 21–40 years (Table 1).

**Table 1:** Age group of the patients

Age Group	Frequency	Percentage
< 20 Years	19	12.4
21 to 40 Years	65	42.5
41 to 60 Years	63	41.2
61 to 80 Years	6	3.9
Total	153	100

**Fig. 1:**
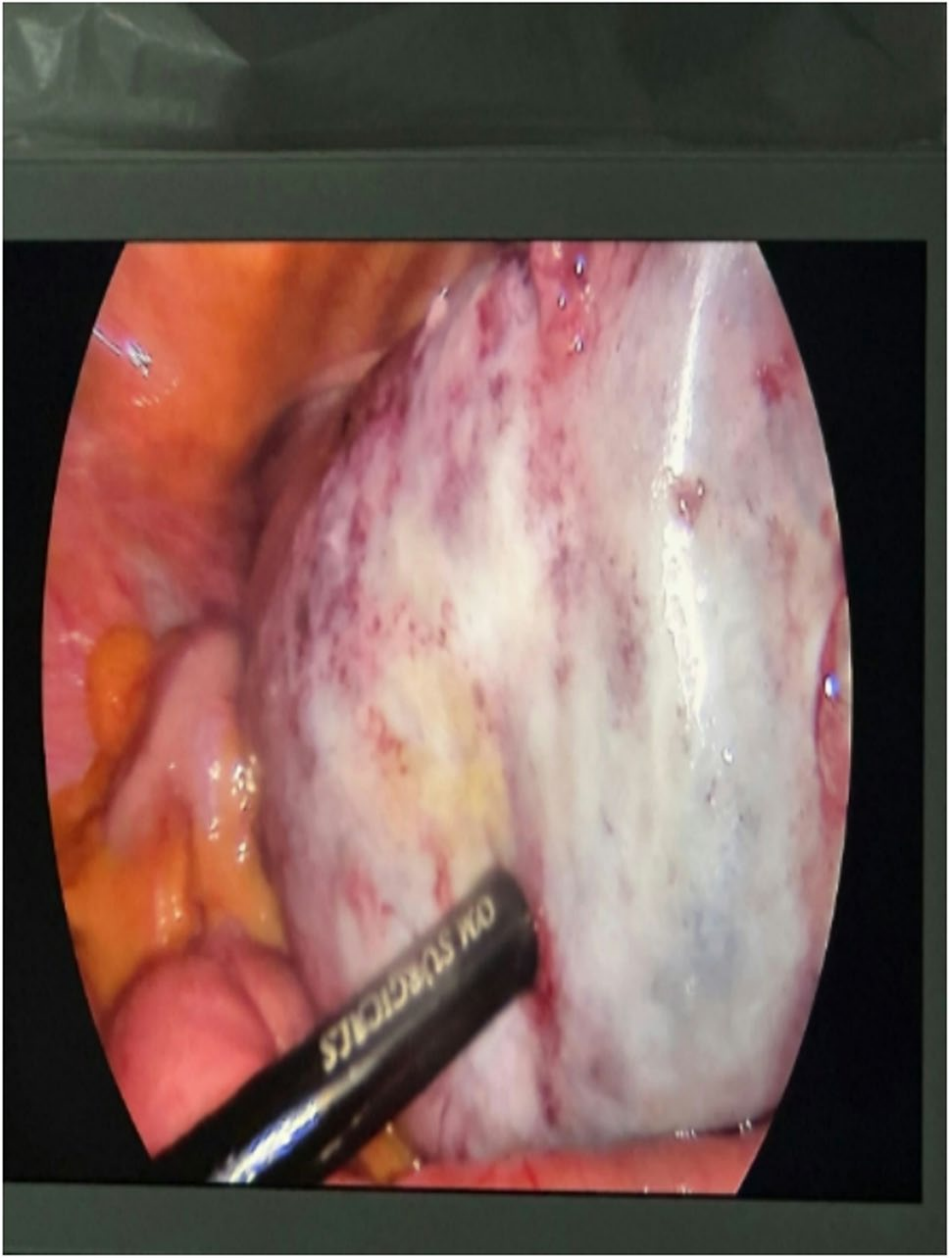
Ovarian Cyst

**Fig. 2:**
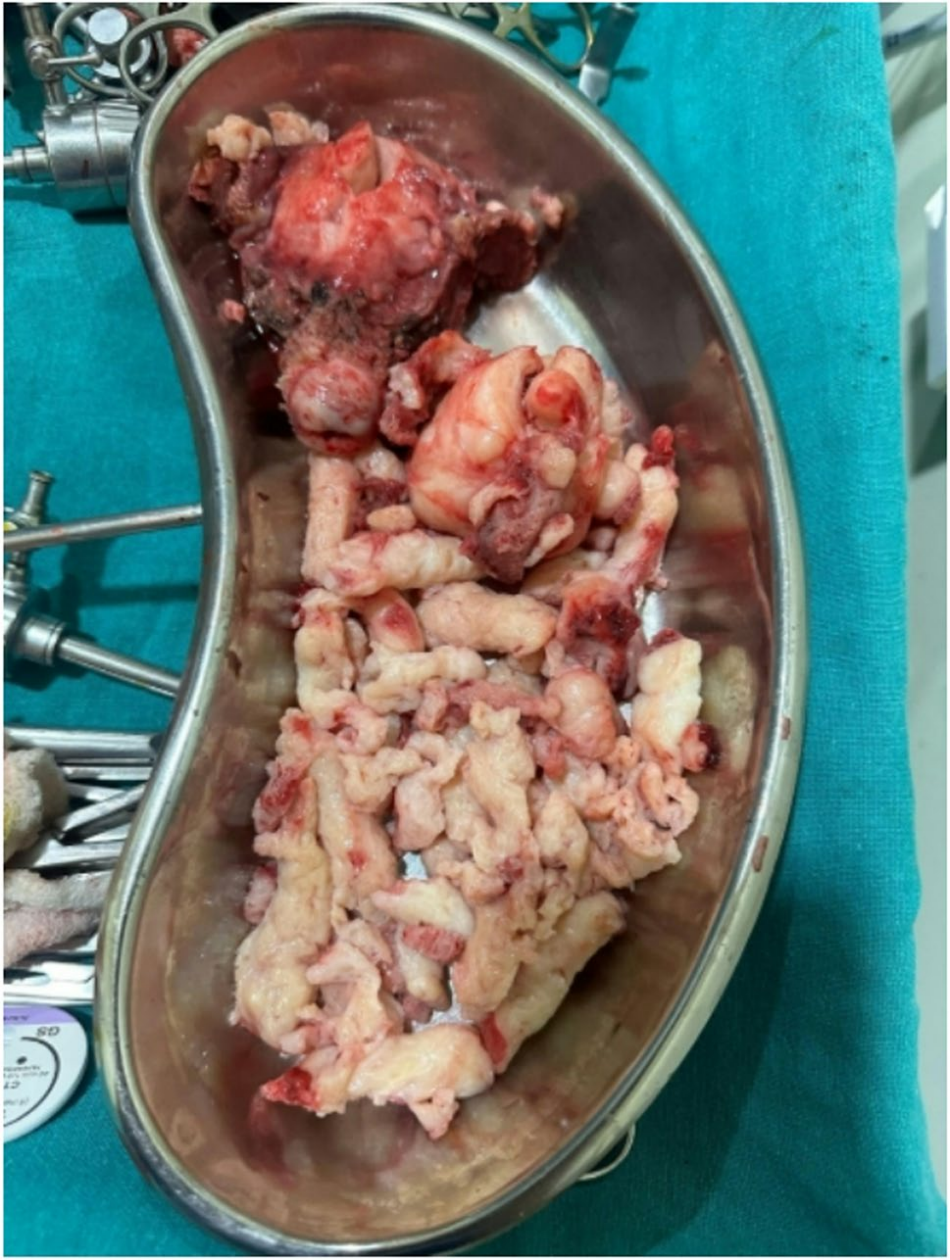
Uterine fibroid

**Fig. 3:**
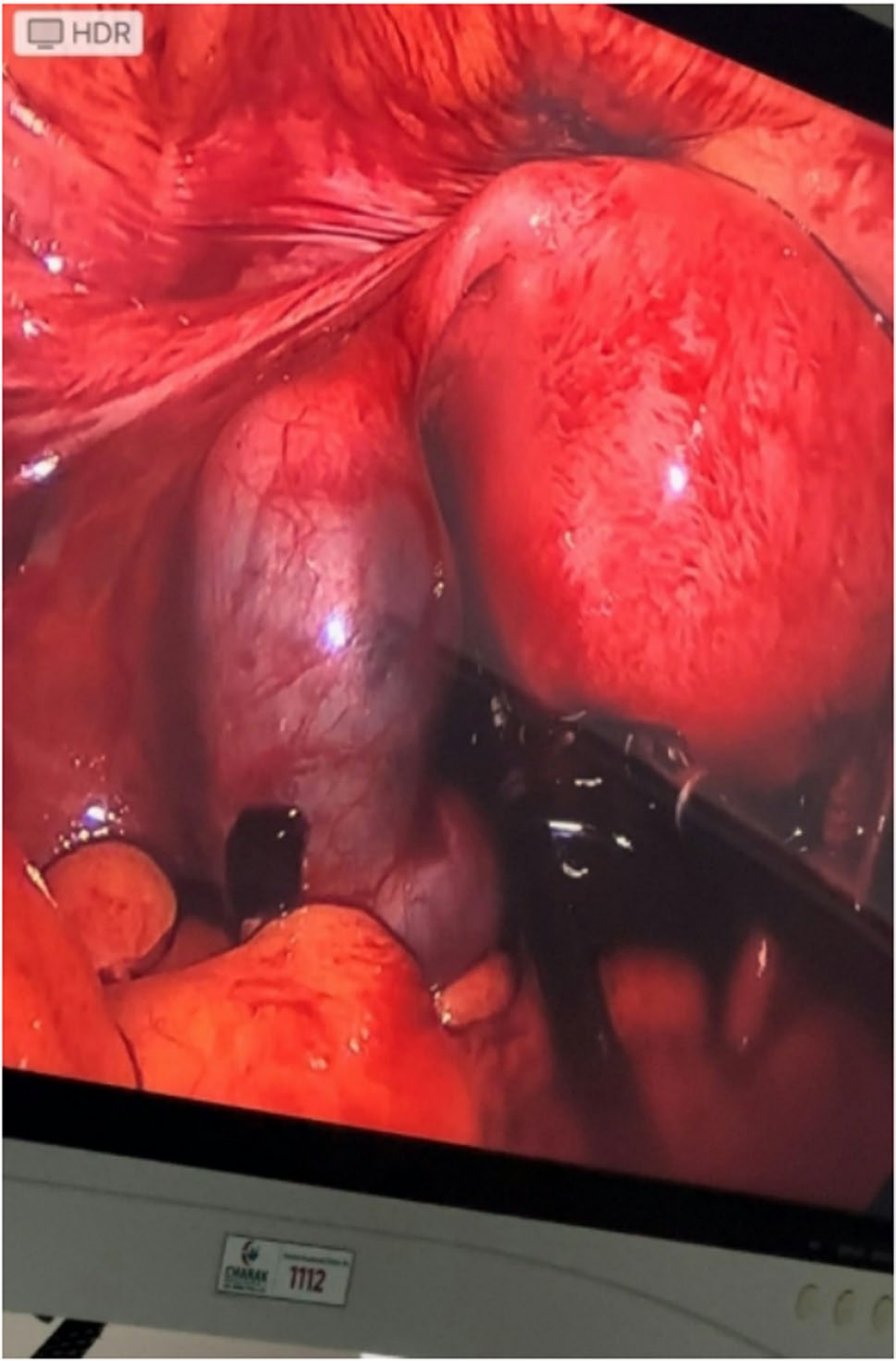
Tubal ectopic

Among these, ovarian cystectomies emerged as the most common procedure, accounting for 62.1% of cases(Fig. 1). Total laparoscopic hysterectomy (TLH) constituted 26.8% of surgeries (Fig. 2), primarily performed for fibroids. Diagnostic laparoscopy for chronic pelvic pain was conducted in 1.3% of cases (Fig. 3) (Table 2).

**Table 2:** Different laparoscopic procedures performed

S.*N*.	Laparoscopic procedure	Number	Percentage
1.	Ovarian Cystectomy	95	62.1%
2.	Total Laparoscopic Hysterectomy	41	26.8%
3.	Salpingectomy for Ectopic Pregnancy	11	7.2%
4.	Myomectomy	3	2%
5.	Diagnostic Laparoscopy	2	1.3%
6.	Tubal Sterilization	1	0.7%
	Total	153	100%

Histopathological evaluation of ovarian cystectomy specimens revealed predominantly benign lesions, including functional cysts, dermoid cysts, and serous or mucinous cystadenomas. No malignant pathology was identified in this series.

Six patients required conversion to laparotomy due to surgical complexities. Five patients had dense adhesion and one had profound intraoperative blood loss which made them not amenable to laparoscopic surgery.

Operative time for various gynecological laparoscopic surgeries ranges from 25 min to 240 min. TLH procedures exhibited significantly longer operative times as compared to other surgeries *P* < 0.001 (Table 3).

**Table 3:** Time taken for various laparoscopic surgeries

Operation	Number	Mean Time (min)	Minimum time (mins)	Maximum time (mins)
Cystectomy	95	88.6 min	26	180
TLH	41	196.3 min	160	240
DL	2	50.0	40	60
Salpingectomy	11	101.82	50	180
Myomectomy	3	150.0	150	150
Tubal Sterilization	1	25	25	25
Total	153		25	240

*TLH* Total laparoscopic hysterectomy, *DL* Diagnostic laparoscopy

*P* value < 0.001

Intraoperative bleeding was significantly higher with TLH ( *P* < 0.001). Minimum blood loss and maximum blood loss during TLH was 30 ml and 220 ml respectively (Table 4). In ruptured ectopic pregnancy blood collected previously at the start of the surgery was subtracted from the total blood loss to calculate the intraoperative loss. Ruptured ectopic pregnancy with mild and moderate peritoneal collection in ultrasound with stable vital parameters were managed laparoscopically. Unstable patients with severe shock and gross peritoneal collection in ultrasound were managed by laparotomy.

**Table 4:** Blood loss during various laparoscopic surgeries

Operation	Number	Mean blood Loss (ml)	Minimum blood loss (ml)	Maximum blood loss (ml)
Cystectomy	95	43.03	30.0	123
TLH	41	97.9	30.0	220
DL	2	21.5	20.0	23.0
Salpingectomy	11	72.9	20.0	200.0
Myomectomy	3	60.0	50.0	70.0
Tubal Sterilization	1	20.0	20.0	20.0
Total	153	59.8		

*TLH* Total laparoscopic hysterectomy, *DL* Diagnostic laparoscopy

*P* < 0.001

Postoperative recovery was rapid, with oral diet initiation occurring within 4 to 6 h and mobilization starting after 24 h. Most of the patients (84.9%) were discharged within 2 days of surgery. Rest stayed for 3 days or more. One patient with TLH had a hospital stay of 10 days.

Postoperative recovery was uneventful in the majority of patients. A total of 10 patients experienced postoperative complications. Fever was the most common complication, observed in 5 patients. Three patients developed port-site wound infections, of which one required secondary suturing. One patient developed paralytic ileus, and one had a minimal vault hematoma.

Most complications were minor and managed conservatively. The overall complication rate was 6.5% (10/153).

Most complications were minor (Clavien–Dindo Grade I–II), including postoperative fever (*n* = 5), wound infection (*n* = 3), paralytic ileus (*n* = 1), and vault hematoma (*n* = 1).

One major complication (Grade III or higher) was observed in a patient undergoing total laparoscopic hysterectomy, who developed pelvic vein thrombosis requiring prolonged hospitalization.

One intraoperative complication of urinary bladder injury was noted encountered due to dense adhesions.

Complication rates were higher in more complex procedures such as total laparoscopic hysterectomy.

## Discussion

This study represents an early institutional experience with the adoption of minimally invasive gynecological surgery in a resource-limited setting. While the benefits of MIS are well established globally, evidence from LMICs remains limited, particularly regarding real-world implementation, learning curves, and system-level challenges. Our findings therefore contribute not by introducing new surgical techniques, but by contextualizing the application of MIS within the constraints and realities of a developing healthcare system.

The findings of this study reflect the early experience of adopting minimally invasive gynecological surgery in a resource-constrained setting. Similar to reports from other low- and middle-income countries, ovarian cystectomy was the most commonly performed procedure, likely due to its relative technical simplicity and high prevalence of benign ovarian pathology.

The absence of malignant pathology in ovarian cystectomies further supports the appropriateness of patient selection criteria for laparoscopic management.

Operative duration in our study, particularly for total laparoscopic hysterectomy, was relatively longer compared to some published studies. This can be attributed to the learning curve associated with MIS, especially during the early phase of implementation. Previous literature has consistently shown that operative time decreases with increasing surgical experience and improved team coordination.

The conversion rate to laparotomy in our study was 3.8%, which is comparable to internationally reported rates of approximately 2–5% in early laparoscopic series. Conversions were primarily due to dense adhesions and intraoperative bleeding, both recognized challenges during the early adoption of laparoscopic techniques.

Although this study did not include a direct comparator group of laparotomy patients, the observed outcomes of early oral intake, rapid mobilization, and short hospital stay are consistent with previous studies demonstrating the advantages of minimally invasive surgery over open procedures.

The introduction of MIS in low-resource settings is frequently challenged by limited availability of advanced laparoscopic equipment, financial constraints, and the learning curve associated with complex procedures such as total laparoscopic hysterectomy. Despite these limitations, our experience demonstrates that safe implementation of MIS is feasible with gradual capacity building and increasing surgical experience.

With increasing experience, there was noticeable improvement in surgical efficiency, case selection, and complication management. This aligns with the established concept of the learning curve in minimally invasive surgery.

### Limitations

This study has several limitations. As a retrospective single-center study, it is subject to selection bias and limited generalizability. The absence of a comparator group restricts direct comparison with laparotomy. The learning curve effect likely influenced operative time and perioperative outcomes, particularly during the early phase of MIS implementation. In addition, small sample sizes in certain subgroups limit the strength of subgroup analyses. Although multivariable regression analysis could provide additional insights into predictors of outcomes, the relatively small sample size and heterogeneity of procedures limited the reliability of such analyses.

### Clinical and public health implications

From a global health perspective, expanding access to minimally invasive surgery in LMICs is increasingly recognized as an important component of improving surgical care. Studies such as ours provide practical insights into how MIS can be introduced, adapted, and expanded in resource-constrained settings.

This study represents an early institutional experience with the adoption of minimally invasive gynecological surgery in a resource-limited setting. While the benefits of MIS are well established globally, evidence from LMICs remains limited, particularly regarding real-world implementation, learning curves, and system-level challenges. Our findings therefore contribute not by introducing new surgical techniques, but by contextualizing the application of MIS within the constraints and realities of a developing healthcare system. The first reported laparoscopic gynecological surgery in Nepal was done in 1971 AD at Paropakar maternity and women’s hospital for tubal sterilization [5]. The findings of this study reflect the early experience of adopting minimally invasive gynecological surgery in a resource-constrained setting. Similar to reports from other low- and middle-income countries (LMICs), ovarian cystectomy was the most commonly performed procedure, likely due to its relative technical simplicity and high prevalence of benign ovarian pathology. Studies from comparable settings have also demonstrated that simpler procedures predominate during the initial phase of MIS adoption, with gradual progression to more complex surgeries such as total laparoscopic hysterectomy.

Laparoscopic gynecological surgery was started in Charak Memorial Hospital in 2019 for Ovarian cyst. The high prevalence of ovarian cystectomies in our study reflects the common occurrence of this condition among Nepalese women, emphasizing the importance of offering minimally invasive treatment options which is similar to study by Baral G et al. [5], Adhikari SP et al. [6] and Ghimire A et al. [7].

In our study, the most common age group undergoing laparoscopic surgeries was 21 to 40 years. In a study done by Pudasaini S et al. [8] ovarian cysts were more commonly seen in the age group 21–30 years. It was suggested due to benign ovarian cyst being higher in low parity and prevalence decreases when the parity increases. Similar findings were noted in cross-sectional evidence from the Dongfeng-Tongi cohort study on parity and risk of ovarian cyst [9].

The average duration for the laparoscopic surgeries varies with the type of surgery, availability of the instruments, and experiences of the surgeon. In our study TLH has significantly longer surgery duration than other surgeries. The average duration for TLH was 196 min which is higher than Tamrakar et al. [10] and Adhikari et al. [6]. It is reported that the operating time for laparoscopic surgery is much longer than open surgery, especially in low and middle-income countries because of the setup [11]. Operative duration in our study, particularly for total laparoscopic hysterectomy, was relatively longer compared to some published studies. This can be attributed to the learning curve associated with MIS, especially in the early phase of implementation. Previous literature has consistently shown that operative time decreases with increasing surgical experience and improved team coordination. The longer operative times observed in our study is consistent with early experiences reported internationally, where operative duration is influenced by the learning curve and resource availability.

Intraoperative blood loss depends on the type of surgeries. It was significantly higher in TLH. Cengiz A et al. [12] had shown that parameters like age, BMI, previous surgical history, presence of fibroids, and uterine size did not affect blood loss. The use of laparoscopy technology and vessel-sealing devices are determining factors in the fate of the surgery [12]. The introduction of MIS in LMIC settings is often challenged by limited access to advanced laparoscopic equipment, inconsistent availability of instruments, and financial constraints. These factors can impact operative efficiency and outcomes, particularly during the early stages of program development. Despite these limitations, our experience demonstrates that safe and effective MIS can be implemented with gradual capacity building and institutional support.

The conversion rate observed in our study (3.8%) is comparable to internationally reported rates of approximately 2–5% in early laparoscopic series. Similarly, complication rates in our cohort were within the range reported in global literature, supporting the safety of MIS even during early implementation phases.

The need for conversion to laparotomy in six cases highlights the importance of surgical skill and expertise in performing laparoscopic procedures, as well as the necessity of having resources available for such conversions when necessary. The swift postoperative recovery and short hospital stays observed in this study further support the benefits of MIS, not only in terms of patient comfort and satisfaction but also in terms of healthcare resource utilization. The conversion rate to laparotomy in our study was 3.8%, which is comparable to early experiences reported in similar settings. Conversions were primarily due to dense adhesions and intraoperative bleeding, which are well-recognized challenges during the initial adoption of laparoscopic techniques. This highlights the importance of appropriate case selection and readiness to convert when necessary to ensure patient safety. With increasing experience, there was noticeable improvement in surgical efficiency, case selection, and complication management. This aligns with the concept of the learning curve in MIS, where surgical proficiency improves over time with repeated practice and skill acquisition. Structured training, mentorship, and accumulation of case volume are critical factors in achieving optimal outcomes.

Most of the patients were discharged within 2 days of surgeries. In a study by Adhikari SP et al. [6], the average postoperative hospital stay was 3 days. In a study by Ghimire et al. [7], the average duration of hospital stay was 1.5 days for diagnostic laparoscopy and 2.5 days for therapeutic laparoscopic surgeries. Similarly Yuen et al. [13] reported a significantly shorter hospital stay as compared to open surgery in the laparoscopy group compared to open surgery.

Although this study did not include a direct comparison group of laparotomy patients, the observed outcomes of early oral intake, rapid mobilization, and short hospital stay are consistent with existing literature demonstrating the advantages of minimally invasive surgery over open procedures. Previous studies have shown that laparoscopy is associated with reduced postoperative pain, decreased blood loss, shorter hospital stay, and faster recovery compared to laparotomy. This supports the growing preference for MIS even in resource-limited settings.

Ten patients had postoperative complications, with fever being the most common issue that was treated conservatively. This is similar to a study by Adhikari SP et al. [6]. In contrast, port site infection was found to be the most common consequence in another study conducted in Nepal [14]. The introduction of minimally invasive surgery in our setting was associated with several challenges. These included limited availability of advanced laparoscopic instruments, a significant learning curve particularly for complex procedures such as total laparoscopic hysterectomy, and longer operative times during the initial phase. Additionally, resource constraints and occasional need for conversion to laparotomy due to dense adhesions or intraoperative bleeding posed practical difficulties. Despite these challenges, outcomes improved with increasing surgical experience, highlighting the feasibility of MIS in resource-constrained environments. The absence of malignant pathology in ovarian cystectomies further supports the appropriateness of patient selection criteria for laparoscopic management.

Our findings mirror the global experience that, despite initial challenges, MIS can be successfully integrated into routine gynecological practice in LMICs with progressive improvement in outcomes. From a global health perspective, expanding access to minimally invasive surgery in LMICs is increasingly recognized as an important component of improving surgical care. Studies such as ours provide practical insights into how MIS can be introduced, adapted, and scaled in resource-constrained settings. These findings may inform similar institutions aiming to establish or expand laparoscopic services despite infrastructural and training limitations.

Although multivariable regression analysis could provide further insights into predictors of outcomes, the relatively small sample size and heterogeneity across procedure types limit the reliability of such analyses in this study. Future larger, prospective studies are needed to explore these associations.

This study has several limitations. As a retrospective single-center study, it is subject to selection bias and limited generalizability. The absence of a comparator group (laparotomy) restricts direct evaluation of relative benefits. The learning curve effect likely influenced operative time and outcomes, particularly during the early phase of MIS implementation. Additionally, small sample sizes in certain subgroups limit the robustness of subgroup analyses. These factors should be considered when interpreting the findings.

Postoperative recovery was uneventful in the majority of patients. Oral diet was initiated within 4–6 h after surgery, and mobilization generally started within the first 24 h. Most patients (84.9%) were discharged within two days of surgery.

The overall complication rate was 6.5%. Most complications were minor (Clavien–Dindo Grade I–II). Fever was the most common postoperative complication, occurring in 5 patients. Three patients developed port-site wound infections, one of whom required secondary suturing. One patient developed paralytic ileus, and one patient developed a minimal vault hematoma. These patients were managed conservatively.

One major complication occurred in a patient undergoing total laparoscopic hysterectomy, who developed pelvic vein thrombosis with impending lateral compartment syndrome requiring prolonged hospitalization. One intraoperative urinary bladder injury occurred due to dense adhesions.

## Conclusion

This study demonstrates that minimally invasive gynecological surgery can be performed safely in a resource-constrained setting, with acceptable complication and conversion rates. However, given the descriptive design and absence of a comparator group, conclusions regarding superiority over open surgery cannot be drawn. Further prospective and comparative studies are needed to strengthen the evidence base. Such evidence is valuable for guiding the expansion of MIS services in similar low- and middle-income healthcare environments.

## Data Availability

The data used and analysed during the current study are available from the corresponding author on reasonable request due to hospital policy.

## Abbreviations

MIS: Minimal Invasive Gynecological Surgery
TLH: Total Laparoscopic Hysterectomy
DL: Diagnostic Laparoscopy
